# Editorial: Immunology at the feto-maternal interface

**DOI:** 10.3389/fimmu.2025.1667375

**Published:** 2025-08-12

**Authors:** Amir-Hassan Zarnani, Mahmood Jeddi-Tehrani, Marie-Pierre Piccinni, Maria Laura Zenclussen

**Affiliations:** ^1^ Department of Immunology, School of Public Health, Tehran University of Medical Sciences, Tehran, Iran; ^2^ Reproductive Immunology Research Center, Avicenna Research Institute, Academic Center for Education, Culture and Research (ACECR), Tehran, Iran; ^3^ Department of Tissue Engineering and Regenerative Medicine, Nanobiotechnology Research Center, Avicenna Research Institute, Academic Center for Education, Culture and Research (ACECR), Tehran, Iran; ^4^ Monoclonal Antibody Research Center, Avicenna Research Institute, Academic Center for Education, Culture and Research (ACECR), Tehran, Iran; ^5^ Department of Experimental and Clinical Medicine, University of Florence, Florence, Italy; ^6^ Instituto de Salud y Ambiente del Litoral (ISAL), Universidad Nacional del Litoral (UNL) - Consejo Nacional de Investigaciones Científicas y Técnicas (CONICET), Facultad de Bioquímica y Ciencias Biológicas (FBCB), UNL, Santa Fe, Argentina

**Keywords:** reproduction, immunoregulation, tolerance, endometrium, placenta, inflammation, decidualization

Reproductive biology serves as the foundation for other biological disciplines, as reproduction has been the key driver of evolution in both simple and complex organisms. Evolutionary success is ultimately measured by biological fitness, which hinges on successful reproduction. Consequently, reproductive sciences have become a major focus of research. In particular, reproductive immunology has gained unprecedented attention, driven by societal industrialization, delayed marriages, and declining fertility rates. Despite advancements in assisted reproductive technologies (ART), the low success rate of embryo transfer remains a major bottleneck in *in vitro* fertilization (IVF). Thus, deciphering the immunological mechanisms of embryo implantation—particularly how maternal immune cells at the pregnancy site foster tolerance to fetal antigens—is critical.

The female reproductive tract undergoes immunological changes from the first encounter with seminal fluid, triggering molecular and cellular adaptations that enhance conception and pregnancy by modulating inflammatory responses and inducing paternal antigen-specific regulatory T cells (Tregs) (1). Eutherian implantation evolved from an ancestral inflammatory response to embryo attachment in therian mammals. This explains the dual role of inflammation in human pregnancy—it is essential for both implantation and labor, yet poses a risk to pregnancy maintenance if uncontrolled (2). Thus, inflammation is neither inherently good nor bad—its effects depend on the stage of pregnancy, where it can be either beneficial or harmful. In this regard, many studies have attempted to clarify how inflammation influences the onset of pregnancy and its possible connection to complications like recurrent miscarriage (3). Indeed, several attempts have been made to take advantage of mechanical injury of endometrium to enhance uterine receptivity (4). Nonetheless, controlling inflammation at the feto-maternal interface is a hallmark of a successful pregnancy and in this context both endometrial and placental-derived factors play a role, as highlighted in the reviews by Khorami-Sarvestani et al. and Joseph et al. Placenta, maternal immune cells, and endometrium interact dynamically to adapt the maternal immune system to the genetically distinct fetus and to control inflammatory reactions towards the fetal antigens. Changes in the placental proteome profile may serve as early diagnostic biomarkers for pregnancy disorders. Intriguingly, placenta and cancer development share striking similarities in metabolic activity, cellular behavior, molecular signatures, signaling pathways, and tissue microenvironment. This overlap has led to the theory of “cancer as ectopic trophoblastic cells”, as pointed out by Khorami-Sarvestani et al. Interestingly, the placenta can independently produce melatonin, reducing reliance on the pineal gland. During pregnancy, melatonin safeguards maternal-fetal health by combating oxidative stress via free radical neutralization and modulating inflammation, thereby preserving placental integrity. It also helps regulate maternal immune responses to accommodate the developing fetus throughout gestation, as described by Joseph et al. While acute inflammation initiates implantation and labor, chronic inflammation—linked to conditions such as endometriosis, and chronic endometritis—can lead to cell senescence, infertility, or miscarriage as reviewed by Ticconi et al.. This is mostly driven by the pro-inflammatory cytokines, IL-6 and IL-1, as shown by Presicce et al. Activation of inflammasome by various factors, including pro-inflammatory cytokines, can trigger pyroptosis, an innate immune response leading to activation of apoptotic pathways and perforation of cell membrane. Pyroptosis is known to be activated in pregnancy-related complications such as congenital Zika virus infection, preterm labor, preeclampsia and intrauterine growth restriction as pointed out by Li et al. During late pregnancy, bacterial infections—particularly group B Streptococcus—in the upper reproductive tract frequently trigger chorioamnionitis and inflammatory responses. These infections are a predominant cause of serious pregnancy complications, notably preterm labor and premature delivery. Obese individuals with higher circulating palmitate levels exhibit higher rates of group B Streptococcus colonization compared to those with normal weight. Notably, palmitate and group B streptococcus synergistically and differentially induce IL-1β from human gestational membranes, which would predispose pregnant people to a greater likelihood of pregnancy complications, as highlighted in the work by Gaddy et al.. Bacteria, viruses and parasites could cross the fetal membranes by different endocytotic mechanisms. Given that the maternal-fetal interface must carefully regulate nutrient uptake through endocytosis while preventing harmful substances and drugs from crossing to support healthy fetal development, it is crucial to understand the precise balance between nutrient transport and protection against pathogens, as pointed out in the review by Fan et al..

For a successful human pregnancy, maternal immune tolerance to the semi-allogeneic fetus is essential. This tolerance is controlled by genetic and epigenetic regulations, as described by Liu et al., and is established through a sophisticated interplay of decidual immune cells, cytokines, chemokines, steroid hormones, adhesion molecules, and immune-regulatory factors from both maternal and fetal sources, operating both before and throughout gestation. The maternal-fetal interface hosts a diverse array of immune cells, including decidual natural killer (dNK) cells, macrophages, T cells, and dendritic cells and a minor population of B cells, and NKT cells. These cells interact with decidual stromal cells and trophoblasts, forming an intricate network of cellular communication. Disruptions in this immunological balance can contribute to pregnancy complications such as recurrent miscarriage, preeclampsia, preterm birth, intrauterine growth restriction, and infections (5). Uterine natural killer (uNK) cells, the most abundant immune cells in the pregnant endometrium, are essential for successful pregnancy. Unlike their cytotoxic peripheral counterparts, uNK cells exhibit unique regulatory properties, specializing in tissue homeostasis, vascular remodeling, and immune tolerance. By clearing senescent cells, they maintain decidualization and uterine receptivity. Functional impairment of uNK cells can disrupt endometrial function, contributing to recurrent pregnancy loss. Regulatory T cells (Tregs) suppress inflammatory responses and promote tolerance by inhibiting effector T cells and secreting anti-inflammatory cytokines, IL-10, TGF-β. Their expansion is crucial for preventing fetal rejection. While the roles of steroid hormones, TGFβ and related signaling molecules in induction of Tregs and pregnancy-related immune regulation have been extensively studied, recent studies highlight the potential involvement of uterine Nodal in this process. Previous studies have characterized the expression dynamics, regulatory mechanisms, and functional role of Nodal - a TGFβ superfamily morphogen - during mouse pregnancy. Recently, it was shown that conditional knockout of Nodal in the mouse female reproductive tract caused significant subfertility associated with dysregulation of key inflammatory cytokines, uterine infiltration of pro-inflammatory macrophages and complete absence of CD4+FOXP3+ regulatory T cells during the preimplantation window, as shown in the work by Yull et al..

While B cells and γδ T cells represent a relatively small proportion of decidual immune cells, their critical contributions to sustaining pregnancy cannot be overlooked. γδ T cells have garnered increasing attention for their potential functions in pregnancy. Recognized for their role in monitoring tissue integrity at barrier sites, these cells are more abundant in the decidua among CD3+ lymphocytes than in peripheral blood. Decidual γδ T cell populations - comprising Vδ1+, Vδ2+, and double-negative (Vδ1-/Vδ2-) subsets - differentially express HLA-specific regulatory receptors including NKG2C, NKG2A, ILT2, and KIR2DL4 and secrete key cytokines (G-CSF, FGF2) and cytotoxic factors (Granulysin, IFN-γ), indicating their dual roles in supporting angiogenesis, placental development and providing antimicrobial protection, as highlighted in the work of Nörenberg et al. B cells play an essential yet understudied role in pregnancy, maintaining a delicate equilibrium between immune tolerance for fetal acceptance and protective immunity against infections. Although their importance is recognized, the precise mechanisms by which B cells influence pregnancy outcomes remain poorly understood. Regulatory B cells (Bregs) and their production of IL-10 are pivotal in sustaining fetal tolerance, illustrating the body’s intricate adaptation to prevent rejection of the semi-allogeneic fetus. Conversely, aberrant autoantibody production by specific B cell subsets reveals the fine line between immune regulation and dysfunction, where disruptions can lead to adverse gestational outcomes, as reviewed in the article by Liu et al..

The endometrial immune system, along with other cells not primarily involved in immune functions, releases numerous soluble and regulatory molecules that play a key role in promoting maternal tolerance during pregnancy. These include but are not limited to galectins, indoleamine 2,3-dioxygenase (IDO), and complement regulatory proteins. During pregnancy, complement protein levels increase, but their activity is balanced by high concentrations of regulatory proteins like factor H, which inhibits the alternative C3 convertase and decay-accelerating factor (DAF/CD55), which prevents C3 convertase formation and suppresses downstream complement activation. When the complement system is dysregulated, it can contribute to complications such as pre-eclampsia and intrauterine growth restriction (6). A recent study showed that in the first trimester human placenta, factor H is largely produced by decidual spiral arteries and syncytiotrophoblasts. Notably, placental expression and plasma levels of factor H in preeclampsia women was found comparatively lower than control counterparts, as described in the research article by Yasmin et al.. The activity of complement proteins is related to the receptor they bind. For example, C5a the most powerful anaphylatoxin, triggers diverse immune responses by binding to two transmembrane receptors, C5aR1 and C5aR2. C5aR1 is known for its pro-inflammatory effects, while, C5aR2 KO mice exhibit fewer implantation sites, along with elevated mRNA levels of pro-inflammatory cytokines, IL-12, IL-18, and IFN-γ at the maternal-fetal interface. Additionally, maternal C5aR2 deficiency leads to reduced uterine NK cell infiltration, suggesting a key regulatory role for this receptor in pregnancy maintenance, as pointed out by Froehlich et al..

While a variety of factors and mediators—acting at both endometrial and systemic levels—work in precise harmony to foster immune tolerance at the feto-maternal interface, the critical immunomodulatory role of endometrial cells has often been underestimated. The endometrium is essential for embryo implantation and development, guiding early embryo attachment, supporting placental formation and invasion, and creating a protective environment for fetal growth. A crucial prerequisite for human embryo implantation is the transformation of endometrial stromal cells (ESCs) into a decidualized state. This process involves complex morphological and functional changes in ESCs, accompanied by significant shifts in their epigenetic, metabolomic, transcriptomic, and proteomic profiles. Disruptions in decidualization have been linked to pregnancy loss (7). Decidual stromal cells (DSCs) play a major role in immune regulation, orchestrating, sustaining, and modulating immune responses. They facilitate the recruitment of peripheral blood NK cells (pbNKs), Th1, and Tc lymphocytes, promote the differentiation of uterine NK (uNK) cells, drive monocytes toward an M2-like phenotype, induce tolerogenic dendritic cells, stimulate regulatory T cell (Treg) development, and promote a type 2 immune bias (8). The pivotal role of decidualization in regulating uterine immune milieu has been recently addressed in CBA/J × DBA/2 mouse model. Although this model has long been used to support classical immune tolerance theories during pregnancy and with this notion in mind, a variety of immunomodulators including Tacrolimus has been used to alleviate abortion in this model, like in the article by Meng et al., new findings indicate that immune imbalance is not the primary cause of fetal loss. Instead, the authors demonstrated that impaired endometrial decidualization likely triggers immune dysregulation at the maternal-fetal interface, ultimately resulting in pregnancy failure, as shown by Zarnani et al..

Alongside the well-established role of immunoregulation in fostering maternal tolerance to the feto-placental unit, immune dysregulation has also been widely investigated in the context of pregnancy complications. Pregnancy-related disorders arise from diverse and intricate pathogenic mechanisms, with immune-mediated processes playing a central role in nearly all cases. Understanding the precise contribution of the immune system in these conditions is therefore critical. While past research primarily relied on reductionist methods—examining isolated or limited disease-associated factors—advances in high-throughput technologies now allow for a comprehensive, systems-level approach. This shift enables more precise immunodiagnosis and targeted therapies for women with pregnancy-related complications (9). Among these emerging strategies, endometrial immune profiling stands out as a key tool for characterizing the endometrial milieu including immune cells and soluble immunoregulatory proteins before or during pregnancy. This approach focuses on deciphering localized immune responses within the endometrium to uncover their role in reproductive success or pathology. This approach has been the focus of recent papers for preconceptional immunodiagnosis of different diseases including systemic autoimmune diseases (as highlighted by Fierro et al.) and preeclampsia (as demonstrated by Tsuda et al.). It has also been suggested by Lédée et al., that this approach could enhance the overall performance of assisted reproductive therapies.

This Research Topic in Frontiers in Immunology mainly covers fundamental aspects of the immunoregulatory processes within the innate and adaptive immune systems that facilitate successful embryo implantation and placental formation. It also explores the immunopathology that leads to adverse pregnancy outcomes, as detailed in the articles by Ramos et al. and by de la Fuente-Munoz et al.. Furthermore, it sheds light on the emerging technologies for the immunodiagnosis of pregnancy-related disorders.
